# Genetics of energy allocation to growth in Angus cattle: trait derivation, model comparison, and relationships with feed efficiency and body composition traits

**DOI:** 10.1186/s12711-026-01102-4

**Published:** 2026-10-31

**Authors:** Qianqian Huang, Nicolas C. Friggens, Hinayah R. de Oliveira, Allan P. Schinckel, Guilherme J. M. Rosa, Andre Garcia, Kelli Retallick, Miguel H. A. Santana, Luiz F. Brito

**Affiliations:** 1 Department of Animal Sciences, Purdue University, West Lafayette, IN USA; 2 UMR PEGASE, INRAE, Institut Agro Rennes, Saint-Gilles, 35590 France; 3 Department of Animal and Dairy Sciences, University of Wisconsin– Madison, Madison, WI USA; 4 American Angus Association, Angus Genetics Inc, Saint Joseph, MO USA; 5 School of Animal Science and Food Engineering, University of São Paulo, Pirassununga, SP Brazil

## Abstract

**Background:**

Improving feed efficiency while understanding resource-allocation trade-offs remains a challenge in livestock breeding. We defined an energy allocation coefficient to growth (*α*) as the ratio of estimated net energy retained in growth to net energy available after accounting for predicted maintenance requirements and investigated its genetic background in American Angus cattle. We compared a two-step (TS) approach, treating *α* as a derived pseudo-phenotype in linear animal models, with a one-step (OS) approach, jointly estimating breeding values and variance components through a nonlinear energy-balance model. Genetic parameters were estimated using pedigree- and genomic-based models, and genetic relationships with growth, feed efficiency, and body composition traits were evaluated.

**Results:**

The derived *α* averaged 0.77 ± 0.28 and ranged from 0.07 to 3.10. Log(*α*) was heritable (h² ≈ 0.13–0.20), with highly consistent breeding value rankings across approaches (*ρ* > 0.94). Genetic correlations were 0.63 ± 0.03 with average daily gain, −​0.80 ± 0.02 with feed conversion ratio, −​0.54 ± 0.03 with residual feed intake, and 0.61 ± 0.03 with residual average daily gain, indicating substantial but incomplete genetic overlap with conventional growth and feed efficiency traits. The genetic correlation with dry matter intake was weak (−​0.14 ± 0.04) and close to zero with body composition traits (−​0.13 to 0.04).

**Conclusions:**

The *α* is a derived indicator of relative energy allocation toward growth, calculated using a nutritional energy-balance framework, and contains additive genetic variation in American Angus cattle. Despite modest differences in variance partitioning between TS and OS, the underlying genetic signal was largely consistent across model formulations. The TS framework is compatible with standard genetic evaluation procedures and provides a practical approach for genetic analysis of this derived coefficient. The incomplete genetic overlap between *α* and conventional feed efficiency traits supports further evaluation of *α* as a complementary indicator of efficiency from a resource-allocation perspective. Future work should evaluate its relationships with health, reproduction, and resilience-related traits to determine whether *α* provides additional information for investigating resource-allocation trade-offs and future breeding applications.

**Supplementary Information:**

The online version contains supplementary material available at https://doi.org/10.1186/s12711-026-01102-4.

## Background

Animal performance depends on acquisition and allocation of resources (e.g., energy, nutrients) among competing physiological functions, including maintenance, growth and other productive outputs (e.g., milk yield), reproduction, immune responses, thermoregulation, and physical activity. This energy allocation perspective is a cornerstone of life-history theory, which predicts that when the total pool of available resources is constrained, increased allocation to one function is expected to reduce resources available for others, potentially generating trade-offs among traits [1, 2]. Importantly, such trade-offs are not always empirically observed, because variation in resource acquisition among individuals may mask underlying allocation trade-offs, whereby individuals with greater resource acquisition can invest more in multiple functions simultaneously [1, 3]. While this framework was originally developed to interpret evolutionary patterns in wild populations, it has been increasingly applied in livestock systems to understand correlated responses to selection and the emergence of unfavorable side effects associated with sustained genetic improvement in productivity traits [4, 5].

Under variable commercial production environments, substantial between-animal variation in growth rate and feed efficiency has been consistently observed, even among animals managed under broadly similar nutritional and management conditions [6]. Variation in feed efficiency cannot be fully explained by differences in feed intake (FI), body size, or growth rate alone. Indeed, after accounting for expected intake requirements associated with maintenance and production, animals still differ in efficiency, as captured by residual feed intake (RFI) and related metrics [7, 8]. These differences have been discussed in the context of heterogeneity in intrinsic energy use and resource partitioning. Animals allocating more metabolizable (ME) or net energy (NE) toward growth may achieve faster weight gain or improved efficiency, whereas energy use associated with other biological functions, such as maintenance, activity, thermoregulation, and immune responses, may reduce the energy available for growth [2, 4, 9]. Quantifying energy allocation toward growth, rather than relying solely on outcome-based efficiency traits, could provide a complementary perspective on biological efficiency and a basis for investigating potential trade-offs between productivity and other biological functions.

Directly measuring how ME or NE is allocated among physiological functions (e.g., growth, maintenance, activity, thermoregulation, immune response) remains difficult in large-scale field settings. Nevertheless, the increasing deployment of electronic FI recording technologies has expanded the availability of individual FI data. Automated feeding systems can record FI at daily resolution or on a per-visit basis. However, in beef cattle research and genetic evaluations, FI and growth are commonly summarized into standardized test-period phenotypes, such as average daily dry matter intake (DMI) and average daily gain (ADG), following industry testing guidelines that specify adaptation periods and minimum test durations [10, 11].

Nutritional energy systems provide a quantitative framework for relating FI, predicted maintenance requirements, and energy retained in production. In beef cattle, these systems allow the energy represented by growth to be evaluated relative to the estimated energy available after maintenance requirements are accounted for [12], providing a basis for deriving energy-allocation indicators from routinely recorded FI and body weight (BW) data.

In animal breeding, intermediate or derived traits are often constructed from observed data using equations or statistical adjustments and subsequently analyzed using linear mixed models. Well-known examples include RFI and feed conversion ratio (FCR) [7, 13]. However, traits derived through ratios or nonlinear transformations can exhibit undesirable statistical properties and interpretational challenges, and ratio traits have been shown to complicate selection responses because genetic change depends on the covariance structure of the component traits [14]. In addition, uncertainty introduced during the derivation of such traits is typically not accounted for in subsequent mixed model analyses, which can affect the partitioning of residual and additive genetic variation and, consequently, genetic parameter estimates. These considerations motivate evaluating the robustness of derived phenotypes and exploring complementary approaches that enable the estimation of genetic parameters directly without the need for pseudo-phenotype derivation.

Here, we define the energy allocation coefficient to growth (*α*) as the ratio of estimated NE retained in growth to estimated NE available for growth after accounting for predicted maintenance requirements. Thus, *α* is a derived indicator of relative energy allocation toward growth, calculated using a nutritional energy-balance framework, rather than a direct measurement of physiological energy partitioning. Higher *α* values indicate greater estimated energy retained in growth relative to the estimated energy available for growth under the assumptions of the adopted energy system. Two complementary strategies were applied to estimate genetic parameters of *α*. First, a two-step (TS) approach consists of deriving a pseudo-phenotype from the nutritional energy-balance framework and subsequently analyzing it using a linear mixed model. Second, a one-step (OS) approach allows direct modeling of empty body weight gain (EBWG) as a nonlinear function of *α* within a mixed-model framework, enabling the joint estimation of breeding values and variance components without constructing pseudo-phenotypes.

Some genetic overlap with conventional growth and feed efficiency traits was expected, as *α* is derived from intake- and growth-related energy components. However, the extent of this overlap and whether *α* is genetically equivalent to these traits was unknown. Accordingly, we investigated whether an energy-allocation coefficient derived from routinely available intake and growth data using a nutritional energy-balance framework contains additive genetic variation, and to what extent its genetic background overlaps with conventional growth and feed efficiency traits. Therefore, the objectives of this study were to: (1) quantify the genetic basis of *α* through (co)variance component and genetic parameter estimation using the complementary TS and OS approaches; and (2) characterize the genetic relationships of *α* with growth, feed efficiency, and body composition traits in American Angus cattle.

## Methods

### Study population and data sources

Phenotypic and pedigree data were provided by the American Angus Association (AAA; Saint Joseph, MO, USA). The initial dataset included 39,071 purebred Angus cattle, comprising 27,882 bulls, 3964 steers, and 7225 heifers, tested between March 1991 and January 2025. Animals were evaluated in 903 FI test contemporary groups (CG) across 179 farms located in 32 U.S. states (Additional file 1 Figure S1). The average test duration was 73.0 ± 21.2 days. CG were defined by test cohort and feeding station and were separated by sex and age under the standardized FI testing protocol. The AAA FI testing guidelines specify a conditioning period before testing, followed by *ad libitum* access to the final test diet during the test period. Animals within the same test are expected to receive the same diet, with diet composition maintained constant throughout the test period. Farm- and test-specific diet composition, however, was not available in the dataset used in this study. The pedigree contained 132,465 individuals, including 20,637 sires and 91,175 dams. Among these, 3525 sires and 24,660 dams had progeny records in the phenotypic dataset.

Phenotypic records included DMI (kg/d), BW (kg), ultrasound ribeye area (UREA; cm²), ultrasound rib fat thickness (UFATrib; cm), ultrasound rump fat thickness (UFATrump; cm), ultrasound intramuscular fat percentage (UIMF; %), and yearling hip height (YHP; cm). Individual FI was recorded using electronic FI systems, and the records provided by the AAA were expressed on a dry matter basis as average daily DMI, consistent with the testing guidelines for improving comparability across tests. Only test-period average DMI was available in the dataset used for this study, rather than daily DMI records. BW was recorded at the beginning and end of the feeding test. Ultrasound traits were measured near the end of the feeding period, at a mean age of 375.0 ± 27.5 days.

Animals had been genotyped using different single nucleotide polymorphism (SNP) arrays over time, and genotypes were imputed using FImpute software [15] to a common marker density of 54,609 SNPs as part of the official genomic evaluation pipeline implemented by Angus Genetics Inc. Genomic quality control was subsequently applied to the imputed genotypes.

Meteorological data were obtained from NASA POWER (https://power.larc.nasa.gov/) using the geographic coordinates of each feeding test station. Mean environmental temperature was calculated over each animal’s feeding test period and was subsequently used to estimate individual NE requirements for maintenance.

### Data quality control

Quality control was applied to phenotypic and genomic data before subsequent analyses. For phenotypic records, ADG was calculated as the change in BW from the beginning to the end of the feeding test divided by test duration. Records outside predefined ranges based on biological plausibility and measurement reliability were excluded: DMI, 2–20 kg/d; ADG, 0.30–2.50 kg/d; feeding test duration, 45–135 days; UFATrib and UFATrump, 0.10–2.50 cm; UIMF, 1–10%; and UREA, 40–120 cm². Animals with missing DMI or ADG records were excluded. Animals born before 2005 were also removed because of sparse representation and less standardized FI recording in earlier years. CG with fewer than five animals were excluded. After phenotypic quality control, 35,772 animals remained, including 25,987 bulls, 2886 steers, and 6899 heifers, distributed across 851 CG. Descriptive statistics of the retained CG are presented in Table 1.

Genomic quality control was applied to the imputed dataset. SNPs were excluded if they had a minor allele frequency below 0.05, a call rate below 0.90, were located on non-autosomal chromosomes, or had duplicated or unknown genomic positions. Animals with a genotype call rate below 0.90 were also excluded. After quality control, 39,412 autosomal SNPs and 14,090 genotyped animals remained for subsequent genomic analyses.

**Table 1:** Descriptive statistics for feeding test contemporary groups in American Angus cattle after data editing and quality control

Variable	Value
Total number of contemporary groups	851
Number of groups with only bulls	661
Number of groups with only steers	29
Number of groups with only heifers	161
Average number of animals in each group	42.0 ± 46.2
Distribution of groups by seasons ^a^	
Spring	119
Summer	179
Fall	205
Winter	348

^a^ Seasons were defined based on the midpoint of the feeding test period for each contemporary group. The midpoint was calculated at the individual level as the average of the start and end dates and then averaged within contemporary groups. Spring = March–May, Summer = June–August, Fall = September–November, and Winter = December–February

### Derived feed efficiency traits

Using the quality-controlled phenotypic records, FCR was calculated as DMI divided by ADG. As a ratio trait, FCR may exhibit extreme values even when DMI and ADG individually fall within their predefined ranges. Therefore, FCR values outside the biologically plausible range of 2 to 15 were set as missing. Mid-test BW was calculated as the average of BW recorded at the beginning and end of the feeding test, and mid-test metabolic body weight (MBW) was defined as mid-test BW raised to the power of 0.75 [16]. RFI and residual average daily gain (RADG) were obtained as residuals from separate multiple linear regression models fitted to the quality-controlled dataset [7]:

$$\:{\mathrm{D}\mathrm{M}\mathrm{I}}_{i}={a}_{0}+{a}_{1}{\mathrm{A}\mathrm{D}\mathrm{G}}_{i}+{a}_{2}{\mathrm{M}\mathrm{B}\mathrm{W}}_{i}+{a}_{3}{\mathrm{A}\mathrm{g}\mathrm{e}}_{z,i}+{e}_{\mathrm{D}\mathrm{M}\mathrm{I},i},$$

$$\:{\mathrm{A}\mathrm{D}\mathrm{G}}_{i}={b}_{0}+{b}_{1}{\mathrm{D}\mathrm{M}\mathrm{I}}_{i}+{b}_{2}{\mathrm{M}\mathrm{B}\mathrm{W}}_{i}+{b}_{3}{\mathrm{A}\mathrm{g}\mathrm{e}}_{z,i}+{e}_{\mathrm{A}\mathrm{D}\mathrm{G},i},$$

where * i* denotes animal; $\:{a}_{0}$ and $\:{b}_{0}$ are intercepts; $\:{a}_{1}$–$\:{a}_{3}$ and $\:{b}_{1}$–$\:{b}_{3}$ are regression coefficients; $\:{\mathrm{M}\mathrm{B}\mathrm{W}}_{i}$ is the mid-test metabolic BW of animal * i*; and $\:{\mathrm{A}\mathrm{g}\mathrm{e}}_{z,i}$ is the standardized mid-test age. The residuals $\:{e}_{\mathrm{D}\mathrm{M}\mathrm{I},i}$ and $\:{e}_{\mathrm{A}\mathrm{D}\mathrm{G},i}$ were defined as RFI and RADG, respectively.

### Derivation of the energy allocation coefficient

The *α* was derived for animal * i* as the ratio of estimated NE retained in growth to estimated NE available for growth after accounting for maintenance requirements:

$$\:{\alpha\:}_{i}=\frac{{\mathrm{N}\mathrm{E}\mathrm{g}}_{i}}{{k}_{g}{S}_{i}},$$

where $\:{\mathrm{N}\mathrm{E}\mathrm{g}}_{i}$ is the estimated daily NE retained in growth, $\:{S}_{i}\:$is the ME remaining after accounting for predicted maintenance requirements, and $\:{k}_{g}$ is the partial efficiency of converting ME to NE for growth. Thus, $\:{k}_{g}{S}_{i}$ represents the estimated NE available for growth. The components of this equation were calculated as described below.

Metabolizable energy intake (MEI, Mcal/d) was calculated from DMI using an assumed constant metabolizable energy density (MED) of 3.0 Mcal/kg DM [12, 17]:

$$\:{\mathrm{M}\mathrm{E}\mathrm{I}}_{i}={\mathrm{D}\mathrm{M}\mathrm{I}}_{i}\times\:\mathrm{M}\mathrm{E}\mathrm{D}.$$

Farm- and test-specific diet composition and energy density were not available in the dataset; therefore, the same MED was assumed across animals. The partial efficiencies of ME utilization were set to $\:{k}_{m}=0.70$ for maintenance and $\:{k}_{g}=0.50$ for growth, consistent with commonly used approximations for high energy beef cattle diets [12, 18].

Shrunk body weight ($\:{\mathrm{S}\mathrm{B}\mathrm{W}}_{i}$) was calculated from mid-test BW as:

$$\:{\mathrm{S}\mathrm{B}\mathrm{W}}_{i}=0.96\times\:{\mathrm{B}\mathrm{W}\mathrm{m}\mathrm{i}\mathrm{d}}_{i}.$$

The individual NE requirement for maintenance ($\:{\mathrm{N}\mathrm{E}\mathrm{m}}_{i}^{req}$, Mcal/d) was estimated following the National Academies of Sciences, Engineering, and Medicine [12]:

$$\:{\mathrm{N}\mathrm{E}\mathrm{m}}_{i}^{req}={\mathrm{S}\mathrm{B}\mathrm{W}}_{i}^{0.75}\left[0.077+0.0007\left(20-{T}_{i}\right)\right],$$

where $\:{T}_{i}$ is the mean environmental temperature (°C) during the feeding test of animal * i*. The corresponding ME requirement for maintenance was obtained by dividing $\:NE{m}_{i}^{req}$ by $\:{k}_{m}$. The ME remaining after accounting for this requirement was then calculated as:

$$\:{S}_{i}={\mathrm{M}\mathrm{E}\mathrm{I}}_{i}-\frac{{\mathrm{N}\mathrm{E}\mathrm{m}}_{i}^{req}}{{k}_{m}}.$$

The estimated NE available for growth was therefore $\:{k}_{g}{S}_{i}$.

Daily NE retained in growth was estimated using the comparative slaughter-based retained energy equation adopted in beef cattle energy systems [12, 13]:

$$\:{\mathrm{N}\mathrm{E}\mathrm{g}}_{i}=0.0635\times\:{\mathrm{E}\mathrm{B}\mathrm{W}}_{i}^{0.75}\times\:{\mathrm{E}\mathrm{B}\mathrm{W}\mathrm{G}}_{i}^{1.097},$$

where $\:{\mathrm{N}\mathrm{E}\mathrm{g}}_{i}$ is expressed in Mcal/d, $\:{\mathrm{E}\mathrm{B}\mathrm{W}}_{i}$ is empty BW (kg), and $\:{\mathrm{E}\mathrm{B}\mathrm{W}\mathrm{G}}_{i}$ is empty BW gain (kg/d). Empty BW and EBWG were calculated as $\:0.855\times\:{\mathrm{B}\mathrm{W}\mathrm{m}\mathrm{i}\mathrm{d}}_{i}$ and $\:0.855\times\:{\mathrm{A}\mathrm{D}\mathrm{G}}_{i}$, respectively. Thus, the equation allows the estimated energy retained in growth to vary with both body size and rate of gain rather than assuming a constant energetic value per unit of gain.

In summary, MED, $\:{k}_{m}$, and $\:{k}_{g}$ were assumed constant, whereas $\:ME{I}_{i}$, $\:NE{m}_{i}^{req}$, $\:{S}_{i}$, $\:EB{W}_{i}$, $\:EBW{G}_{i}$, $\:NE{g}_{i}$, and $\:{\alpha\:}_{i}\:$were calculated for each animal.

To avoid undefined or numerically unstable *α* estimates when estimated NE available for growth was non-positive or approached zero, $\:{\alpha\:}_{i}\:$was set to missing when its denominator ($\:{k}_{g}{S}_{i}$) was less than or equal to a threshold defined as the 1st percentile of the absolute distribution of estimated NE available for growth. A total of 35,276 animals had valid $\:\alpha\:$ phenotypes. The derived $\:\alpha\:$ was natural log-transformed before genetic analyses to improve normality and stabilize residual variance.

### Genetic analyses and model comparison

Genetic parameters of log-transformed $\:\alpha\:$ were estimated using two modeling approaches: TS approach and OS approach. The TS approach treated the derived log($\:\alpha\:$) phenotype as the response variable in a linear animal model, whereas the OS approach estimated genetic effects on $\:\alpha\:$ directly within the nonlinear energy-balance model. CG was evaluated as either a fixed or random effect in both approaches. For the TS approach, genetic analyses were conducted using either the pedigree-based numerator relationship matrix ($\:\mathbf{A}$) or the single-step relationship matrix ($\:\mathbf{H}$), which combines pedigree and genomic information. The OS approach was fitted using the $\:{\mathbf{A}}^{-1}$ matrix only due to the computational demands of incorporating the denser $\:{\mathbf{H}}^{-1}$ structure into the nonlinear model.

Six model specifications were therefore compared: TSFH, TSRH, TSFA, TSRA, OSFA, and OSRA. The first two letters indicate the modeling approach (TS or OS), the third letter indicates whether CG was fitted as fixed (F) or random (R), and the final letter denotes the relationship matrix used for genetic effects ($\:\mathbf{A}$ or $\:\mathbf{H}$). For example, TSFH represents the TS approach with fixed CG and the $\:\mathbf{H}$ matrix, whereas OSRA represents the OS approach with random CG and the $\:\mathbf{A}$ matrix.

#### Two-step (TS) Approach

In the TS approach, log($\:\alpha\:$) was analyzed as a single trait using the following animal model:

$$\:\mathbf{y}=\mathbf{X}\mathbf{b}+\mathbf{Z}\mathbf{a}+\mathbf{W}\mathbf{c}+\mathbf{e},$$

where **y** is the vector of log($\:\alpha\:$) phenotypes; **b** is the vector of fixed effects; $\:\mathbf{a}$ is the vector of additive genetic effects; $\:\mathbf{c}\:$is the vector of random CG effects; and $\:\mathbf{e}$ is the vector of residuals. The matrices $\:\mathbf{X}$, $\:\mathbf{Z}$, and $\:\mathbf{W}$ are the corresponding incidence matrices. Standardized mid-test age was fitted as a linear covariate in all models. CG was included in $\:\mathbf{b}$ when fitted as a fixed effect. For random-CG models, CG was instead included in $\:\mathbf{c}$, with $\:\mathbf{c}\sim\:N\left(0,\mathbf{I}{\sigma\:}_{c}^{2}\right).$ Additive genetic effects were assumed to follow $\:\mathbf{a}\sim\:N\left(0,\mathbf{K}{\sigma\:}_{a}^{2}\right),\:$where $\:\mathbf{K}$ represents either the pedigree-based relationship matrix ($\:\mathbf{A}$) or the single-step relationship matrix ($\:\mathbf{H}$). Residuals were assumed as $\:\mathbf{e}\sim\:N(0,\mathbf{I}{\sigma\:}_{e}^{2})$. Random CG, additive genetic effects, and residual effects were assumed to be mutually independent. Variance components were estimated using the Average Information Restricted Maximum Likelihood (AI-REML) algorithm implemented in BLUPF90+ [19].

#### Relationship matrices

For pedigree-based analyses, additive genetic relationships among animals were represented by the numerator relationship matrix ($\:\mathbf{A}$). For single-step genomic analyses, pedigree and genomic information were combined through the $\:\mathbf{H}$ matrix. Its inverse was constructed as [20]:

$$\:{\mathbf{H}}^{-1}={\mathbf{A}}^{-1}+\left[\begin{array}{cc}0&\:0\\\:0&\:({\mathbf{G}}_{\omega\:}^{-1}-{\mathbf{A}}_{22}^{-1})\end{array}\right],$$

where $\:{\mathbf{A}}^{-1}$ is the inverse of the pedigree-based relationship matrix, $\:{\mathbf{A}}_{22}^{-1}$ is the inverse of the pedigree relationship submatrix for genotyped animals, and $\:{\mathbf{G}}_{\omega\:}^{-1}$ is the inverse of the blended genomic relationship matrix. The genomic relationship matrix **G** was constructed following Method 1 of VanRaden (2008) [21] as $\:\mathbf{G}={\mathbf{Z}\mathbf{Z}}^{{\prime\:}}/(2\sum\:{p}_{l}(1-{p}_{l}\left)\right)$, where **Z** is the centered marker genotype matrix and $\:{p}_{l}$ is the observed allele frequency of the * l*-th SNP estimated from genotyped animals. To ensure compatibility between **G** and $\:{\mathbf{A}}_{22}$ and numerical invertibility, **G** was blended as $\:{\mathbf{G}}_{\omega\:}=0.95{\hspace{0.17em}}\mathbf{G}+0.05{\hspace{0.17em}}{\mathbf{A}}_{22}$ following the default parameterization of BLUPF90+ [19].

#### One-step (OS) approach

In the OS approach, EBWG was modeled directly using the same energy-balance relationship used to derive $\:\alpha\:$, without first constructing a pseudo-phenotype. By substituting the retained energy equation into the energy-balance relationship and solving for EBWG, the expected EBWG for animal * i* was expressed as:

$$EBWG_i \sim N\left( \left[ \frac{k_g \, S_i \exp(\eta_i)}{0.0635 \, EBW_i^{0.75}} \right]^{1/1.097},\; \sigma^2_{e,\,EBWG} \right),$$

where $\:{S}_{i}$ is the ME remaining after accounting for predicted maintenance requirements, $\:{k}_{g}$ is the partial efficiency of converting ME to NE for growth, $\:{\eta\:}_{i}$ is the linear predictor for the animal-specific energy allocation coefficient, and $\:{\sigma\:}_{e,EBWG}^{2}$ is the residual variance on the observed EBWG scale.

The animal-specific $\:{\alpha\:}_{i}$ was modeled on the log scale as:

$$\:\mathrm{l}\mathrm{o}\mathrm{g}\left({\alpha\:}_{i}\right)={\eta\:}_{i}=\mu\:+\beta\:Ag{e}_{z,i}+{a}_{i}+{c}_{j},$$

or equivalently,

$$\:{\alpha\:}_{i}=\mathrm{e}\mathrm{x}\mathrm{p}\left({\eta\:}_{i}\right),$$

where $\:\mu\:$ is the overall intercept, $\:\beta\:$ is the regression coefficient for standardized mid-test age, $\:{a}_{i}$ is the additive genetic effect of animal * i*, and $\:{c}_{j}$ is the effect of CG * j*. The log-scale parameterization ensured that fitted values of *α* remained positive and placed additive genetic effects on the same log(*α*) scale used in the TS approach. Thus, the exponential expression is the inverse transformation of the log-scale model rather than an assumption that *α* follows an exponential distribution. Prior to calculating heritability, the observation-specific residual variance was approximated on the $\:\mathrm{l}\mathrm{o}\mathrm{g}\left(\alpha\:\right)$ scale using a first-order delta method [22]. Given the OS expectation $\:{\mu\:}_{i}={\left[{k}_{g}{S}_{i}\mathrm{e}\mathrm{x}\mathrm{p}\left({\eta\:}_{i}\right)/\left(0.0635EB{W}_{i}^{0.75}\right)\right]}^{1/1.097}$, where $\:{\eta\:}_{i}=\mathrm{l}\mathrm{o}\mathrm{g}\left({\alpha\:}_{i}\right)$, the derivative with respect to the latent predictor is $\:\partial\:{\mu\:}_{i}/\partial\:{\eta\:}_{i}={\mu\:}_{i}/1.097$. Thus, the corresponding residual variance on the latent scale was therefore approximated as $\:{\sigma\:}_{e,i,\eta\:}^{2}={\sigma\:}_{e,i,EBWG}^{2}/{\left(\partial\:{\mu\:}_{i}/\partial\:{\eta\:}_{i}\right)}^{2}$. The transformed observation-specific residual variances were averaged across animals to obtain a population-level residual variance for calculation of within-CG heritability.

CG was fitted as either a fixed or random effect. When treated as random, $\:\mathbf{c}\sim\:N\left(0,\mathbf{I}{\sigma\:}_{c}^{2}\right)$. Additive genetic effects were modeled using the pedigree relationship matrix, $\:\mathbf{a}\sim\:N\left(0,\mathbf{A}{\sigma\:}_{a}^{2}\right),\:$ and residuals were assumed to follow $\:\mathbf{e}\sim\:N(0,\mathbf{I}{\sigma\:}_{e}^{2})$.

The OS models were implemented in R (version 4.5.1) [23] using Template Model Builder through the TMB package [24]. Fixed effects and variance components were estimated by maximizing the marginal likelihood using the Laplace approximation. Only pedigree-based OS models were fitted due to the greater computational burden associated with incorporating the single-step relationship structure into the nonlinear likelihood.

#### Heritability and model agreement

Heritability was calculated on a within-CG basis as:

$$\:{\widehat{h}}^{2}=\frac{{\widehat{\sigma\:}}_{a}^{2}}{{\widehat{\sigma\:}}_{a}^{2}+{\widehat{\sigma\:}}_{e}^{2}},$$

where $\:{\widehat{\sigma\:}}_{a}^{2}$ and $\:{\widehat{\sigma\:}}_{e}^{2}$ are the estimated additive genetic and residual variances, respectively. For models in which CG was fitted as a random effect, $\:{\widehat{\sigma\:}}_{c}^{2}$ was excluded from the denominator so that heritability was expressed relative to variation among animals within CG. This within-CG definition provides a directly comparable scale for models in which CG was fitted as either a fixed or random effect.

Theoretical estimated breeding value (EBV) accuracy for animal * i* was calculated as:

$$\:{\mathrm{A}\mathrm{c}\mathrm{c}\mathrm{u}\mathrm{r}\mathrm{a}\mathrm{c}\mathrm{y}}_{i}=\sqrt{1-\frac{{\mathrm{P}\mathrm{E}\mathrm{V}}_{i}}{{\widehat{\sigma\:}}_{a}^{2}}},$$

where $\:{\mathrm{P}\mathrm{E}\mathrm{V}}_{i}$ is the prediction error variance of the EBV for animal * i*, calculated as the squared standard error of the EBV obtained from the corresponding model output, and $\:{\widehat{\sigma\:}}_{a}^{2}$ is the estimated additive genetic variance. Mean EBV accuracy was calculated across animals with phenotypic records.

Agreement among model specifications was evaluated using Spearman rank correlations between EBV and the overlap among animals ranked in the top 25% for EBV. Comparisons were restricted to animals with phenotypic records and EBV accuracy greater than 0.30 in both models being compared. For each model pair, animals were ranked in descending order of EBV, and top-25% overlap was calculated as the proportion of animals appearing in the top quartile under both models.

#### Genetic correlations with feed efficiency and body composition traits

The extent of genetic overlap between log(*α*) and growth, feed efficiency, and body composition traits was evaluated using two-trait animal models. Each model included log(*α*) and one additional trait. For all traits, CG was included as a fixed effect. Additionally, standardized mid-test age was fitted as a linear covariate for ADG, DMI, and FCR, while measurement date was included as fixed covariate for ultrasound traits (UREA, UFATrib, UFATrump, and UIMF). No additional fixed effects were fitted for RFI, RADG, or YHP. Additive genetic effects were modeled using the $\:\mathbf{H}$ matrix, and residual effects were included for both traits. Variance and covariance components were estimated using AI-REML in BLUPF90+ [19].

Genetic correlations were calculated as:

$$\:{\widehat{r}}_{g}=\frac{{\widehat{\sigma\:}}_{a12}}{\sqrt{{\widehat{\sigma\:}}_{a1}^{2}{\widehat{\sigma\:}}_{a2}^{2}}},$$

where $\:{\widehat{\sigma\:}}_{a12}$ is the estimated additive genetic covariance between the two traits, and $\:{\widehat{\sigma\:}}_{a1}^{2}\:$and $\:{\widehat{\sigma\:}}_{a2}^{2}$ are their respective estimated additive genetic variances.

Phenotypic correlations ($\:{r}_{p}$) were calculated as Pearson correlations between log(*α*) and the corresponding observed trait using animals with records for both traits.

## Results

### Descriptive statistics

Descriptive statistics for the analyzed population are shown in Table 2 and Additional file 2: Table S1. Animals were in the growing phase, with an average mid-test age of about 330 ± 49.1 days and a mid-test BW of 414 ± 76.2 kg. They consumed 10.12 ± 2.04 kg of dry matter per day on average and gained 1.58 ± 0.42 kg per day, yielding a FCR of 6.70 ± 1.93. The derived $\:\alpha\:$ averaged 0.77 ± 0.28, ranging from 0.07 to 3.10. As *α* is a derived ratio rather than a bounded proportion, values greater than 1 may occur when estimated NE retained in growth exceeds estimated NE available for growth. Such values may reflect discrepancies between assumed and actual dietary energy density, errors in FI or other input measurements, or individual deviations from the assumptions of the adopted energy system. After log transformation, log(*α*), which was used for subsequent genetic analyses, averaged − 0.34 ± 0.39 and ranged from − 2.60 to 1.13. Ultrasound measurements indicated mean UFATrump and UFATrib of 0.73 ± 0.26 cm and 0.64 ± 0.25 cm, respectively; UREA averaged 77.11 ± 12.67 cm²; and UIMF content was 4.25 ± 1.2%.

**Table 2:** Descriptive statistics for phenotypic records in American Angus cattle

Trait	Unit	*N*	Mean ± SD	Min	Max
Age at the start of feeding test	d	35,772	293.91 ± 50.32	133	432
Age at the end of feeding test	d	35,772	365.63 ± 49.25	202	480
Body weight at the start of feeding test	kg	35,772	357.06 ± 70.88	136.08	698.53
Body weight at the end of feeding test	kg	35,772	470.88 ± 86.68	181.44	814.65
Feeding test duration	d	35,772	71.72 ± 16.67	45	133
Daily dry matter intake (DMI)	kg/d	35,772	10.12 ± 2.04	2.16	19.95
Average daily gain (ADG)	kg/d	35,772	1.58 ± 0.42	0.30	2.50
Feed conversion ratio (FCR)	-	35,302	6.70 ± 1.93	2.01	14.99
Residual feed intake (RFI)	kg/d	35,772	0.00 ± 1.60	-9.12	10.74
Residual average daily gain (RADG)	kg/d	35,772	0.00 ± 0.36	-1.55	1.43
Ultrasound ribeye area (UREA)	cm²	25,683	77.11 ± 12.67	40.65	119.35
Ultrasound rib fat depth (UFATrib)	cm	25,741	0.64 ± 0.25	0.10	2.36
Ultrasound rump fat depth (UFATrump)	cm	25,719	0.73 ± 0.26	0.10	2.39
Ultrasound intramuscular fat (UIMF)	%	25,734	4.25 ± 1.22	1.27	9.97
Yearling hip height (YHP)	cm	14,623	126.0 ± 4.91	93.98	148.59
Energy allocation coefficient ($\:\alpha\:$)	-	35,276	0.77 ± 0.28	0.07	3.10
Log transformed energy allocation coefficient (log$\:\left(\alpha\:\right)$)	-	35,276	-0.34 ± 0.39	-2.60	1.13

### Variance partitioning and model comparison

#### Fixed vs. random contemporary groups

Modeling CG as a fixed or random effect had little impact on estimates of additive genetic and residual variance (Table 3). Under the TS approach with **H**-matrix, shifting from fixed to random CG (TSFH vs. TSRH) partitioned additional between-group variation into the CG variance component ($\:{\widehat{\sigma\:}}_{cg}^{2}=0.177$), while within-CG heritability estimates for log$\:\left(\alpha\:\right)$ remained similar (h^2^ = 0.13 ± 0.01 in TSFH vs. 0.14 ± 0.01 in TSRH). A similar pattern was observed under the OS approach, in which the random-CG model accounted for additional between-group variation ($\:{\widehat{\sigma\:}}_{cg}^{2}=0.107$) but produced comparable within-CG heritability estimates (h^2^ = 0.15 ± 0.01 in OSFA vs. h^2^ = 0.16 ± 0.01 in OSRA). Spearman rank correlations between EBV from the corresponding fixed- and random-CG models were high (*ρ* > 0.92), indicating limited changes in animal ranking across CG specifications.

#### One-step vs. Two-step approach

When TS and OS approaches were compared under the same pedigree-based relationship structure (TSFA vs. OSFA), TS produced a slightly larger estimated additive genetic variance and a slightly smaller estimated residual variance ($\:{\widehat{\sigma\:}}_{a}^{2}=0.010$, $\:{\widehat{\sigma\:}}_{e}^{2}=0.040$) than OS ($\:{\widehat{\sigma\:}}_{a}^{2}=0.008$, $\:{\widehat{\sigma\:}}_{e}^{2}=0.043$), resulting in a higher heritability estimate (0.19 ± 0.01 vs. 0.15 ± 0.01). Despite these differences in variance partitioning, EBV rankings were highly concordant between the two approaches, with a Spearman rank correlation of 0.94 and a top-25% breeding value ranking overlap of 84.3% among animals with phenotypic records.

**Table 3:** Genetic parameters of log transformed energy allocation coefficient (log$\:\left(\alpha\:\right)$) across different models

Model	$\:{\widehat{\boldsymbol{\sigma\:}}}_{\boldsymbol{a}}^{2}$	$\:{\widehat{\boldsymbol{\sigma\:}}}_{\boldsymbol{c}\boldsymbol{g}}^{2}$	$\:{\widehat{\boldsymbol{\sigma\:}}}_{\boldsymbol{e}}^{2}$	h^2^	Accuracy
TSFH	0.007 (0.0004)	-	0.042 (0.0004)	0.13 (0.01)	0.580
TSRH	0.007 (0.0005)	0.178 (0.009)	0.042 (0.0004)	0.14 (0.01)	0.588
OSFA	0.008 (0.0005)	-	0.043 (0.0007)	0.15 (0.01)	0.569
TSFA	0.010 (0.0007)	-	0.040 (0.0006)	0.19 (0.01)	0.577
OSRA	0.008 (0.0005)	0.107 (0.005)	0.043 (0.0007)	0.16 (0.01)	0.580
TSRA	0.010 (0.0007)	0.188 (0.010)	0.039 (0.0006)	0.20 (0.01)	0.583

Model abbreviations: TS, two-step approach; OS, one-step approach; F, fixed contemporary group (CG); R, random CG; H, **H**-matrix (combined pedigree and genomic information); A, **A**-matrix (pedigree only)

$\:{\widehat{\sigma\:}}_{a}^{2}$, estimated additive genetic variance; $\:{\widehat{\sigma\:}}_{cg}^{2}$, estimated random CG variance; $\:{\widehat{\sigma\:}}_{e}^{2}$, estimated residual variance; $\:{h}^{2}$, within-CG heritability; Accuracy, mean EBV accuracy. Standard errors of variance components and $\:{h}^{2}$ are shown in parentheses

Given the overall stability of EBV rankings across model specifications and the availability of genomic information, the TS model with fixed CG and **H**-matrix (TSFH) was selected as the reference model for subsequent analyses.

### Genetic correlations with feed efficiency and body composition traits

Under the reference TSFH model, log(*α*) showed moderate to strong genetic correlations with growth and feed efficiency traits (Table 4). Genetic correlations were positive with ADG (0.63 ± 0.03) and RADG (0.61 ± 0.03), and negative with FCR (− 0.80 ± 0.02) and RFI (− 0.54 ± 0.03). The genetic correlation with DMI was weak (− 0.14 ± 0.04), indicating limited genetic association between log(*α*) and FI in this population. A weaker positive genetic correlation was observed with YHP (0.29 ± 0.04). Overall, the correlations with conventional growth and feed efficiency traits indicate substantial, but incomplete, genetic overlap with log(*α*).

Genetic correlations with body composition traits were generally weak. The log(*α*) was negatively correlated with UFATrib (− 0.12 ± 0.04) and UFATrump (− 0.13 ± 0.04), whereas correlations with UREA (0.04 ± 0.04) and UIMF (− 0.05 ± 0.04) were close to zero. Phenotypic correlations generally followed the same direction as the genetic correlations for growth and feed efficiency traits, with the strongest associations observed for FCR, ADG, RADG, and RFI.

**Table 4:** Genetic parameters and correlations of log transformed energy allocation coefficient ($\log(\alpha)$)with performance, feed efficiency, and body composition traits

Models	$\:{\widehat{\boldsymbol{\sigma\:}}}_{\boldsymbol{a}}^{2}$	$\:{\widehat{\boldsymbol{\sigma\:}}}_{\boldsymbol{e}}^{2}$	h^2^ (trait 1)	h^2^ ($\:\mathbf{l}\mathbf{o}\mathbf{g}\left(\boldsymbol{\alpha\:}\right)$)	*r*_g_	*r*_*p*_
ADG-$\:\mathrm{l}\mathrm{o}\mathrm{g}\left(\alpha\:\right)$	0.008 (0.0006)	0.055 (0.0006)	0.12 (0.01)	0.13 (0.01)	0.63 (0.03)	0.75
FCR-$\:\mathrm{l}\mathrm{o}\mathrm{g}\left(\alpha\:\right)$	0.194 (0.018)	1.908 (0.019)	0.09 (0.01)	0.13 (0.01)	-0.80 (0.02)	-0.85
RFI-$\:\mathrm{l}\mathrm{o}\mathrm{g}\left(\alpha\:\right)$	0.179 (0.011)	0.961 (0.010)	0.16 (0.01)	0.13 (0.01)	-0.54 (0.03)	-0.60
RADG-$\:\mathrm{l}\mathrm{o}\mathrm{g}\left(\alpha\:\right)$	0.005 (0.0004)	0.047 (0.0005)	0.10 (0.01)	0.13 (0.01)	0.61 (0.03)	0.71
DMI-$\:\mathrm{l}\mathrm{o}\mathrm{g}\left(\alpha\:\right)$	0.318 (0.018)	1.316 (0.015)	0.19 (0.01)	0.14 (0.01)	-0.14 (0.04)	-0.11
UFATrump-$\:\mathrm{l}\mathrm{o}\mathrm{g}\left(\alpha\:\right)$	0.016 (0.0006)	0.022 (0.0004)	0.42 (0.01)	0.13 (0.01)	-0.13 (0.04)	0.09
UFATrib-$\:\mathrm{l}\mathrm{o}\mathrm{g}\left(\alpha\:\right)$	0.011 (0.0004)	0.020 (0.0003)	0.35 (0.01)	0.13 (0.01)	-0.12 (0.04)	0.14
UREA-$\:\mathrm{l}\mathrm{o}\mathrm{g}\left(\alpha\:\right)$	18.61 (0.79)	37.13 (0.55)	0.33 (0.01)	0.13 (0.01)	0.04 (0.04)	0.42
UIMF-$\:\mathrm{l}\mathrm{o}\mathrm{g}\left(\alpha\:\right)$	0.248 (0.009)	0.414 (0.006)	0.37 (0.01)	0.13 (0.01)	-0.05 (0.04)	-0.20
YHP-$\:\mathrm{l}\mathrm{o}\mathrm{g}\left(\alpha\:\right)$	4.070	7.834	0.34 (0.02)	0.13 (0.01)	0.29 (0.04)	0.31

$\:{\widehat{\sigma\:}}_{a}^{2}$, estimated additive genetic variance;$\:{\widehat{\sigma\:}}_{e}^{2}$, estimated residual variance;$\:{h}^{2}$, heritability; *r*_*g*_, genetic correlation;$\:{r}_{p}$, phenotypic correlation. Standard errors of variance components,$\:{h}^{2}$and genetic correlations are shown in parentheses

## Discussion

Understanding how animals acquire and allocate resources among physiological functions is relevant to both evolutionary biology and livestock genetic improvement. While genetic selection has historically emphasized outcome-based traits such as growth rate, feed efficiency, or body composition, resource allocation theory provides a conceptual basis for considering how differences in resource acquisition and allocation may contribute to variation in these outcomes. However, translating this conceptual framework into measurable and genetically evaluable traits using large-scale field data remains challenging. Nutritional energy systems provide a quantitative basis for estimating energy associated with maintenance and growth from routinely recorded performance data. Previous studies have attempted to quantify genetic variation in energy utilization using derived energy-related indicators [25, 26], supporting the feasibility of combining energy-based phenotypes with quantitative genetic analyses. In the present study, we used a nutritional energy-balance framework to derive *α* as an indicator of relative energy allocation toward growth and evaluated whether it contains additive genetic variation and how its genetic background overlaps with conventional growth and feed efficiency traits in American Angus cattle.

### Model comparison

#### Fixed vs. random contemporary group (CG)

CG are designed to account for systematic environmental and management differences among animals (e.g., herd-year-season, pen-test day) that would otherwise confound genetic effects, and their specification as fixed or random effects can influence variance partitioning and downstream genetic parameter estimates. In the present study, fitting CG as a random rather than fixed effect did not meaningfully alter estimates of additive genetic variance or residual variance, nor did it substantially affect animal ranking. Heritability estimates reported throughout this study were computed as within-CG heritability, in which $\:{\sigma\:}_{CG}^{2}$ is excluded from the phenotypic variance denominator [27]; under this definition, estimates remained consistent regardless of CG specification. Had heritability been expressed on the total phenotypic variance scale, estimates under random-CG models would have been lower — consistent with the pattern reported by Khorshidi [28] in Hays Converter cattle, where random-CG models increased phenotypic variance and reduced heritability while additive genetic variance remained largely unchanged. Thus, differences between fixed- and random-CG models in the present study primarily reflected alternative partitioning of between-group variation rather than substantial changes in the estimated additive genetic variation.

In beef cattle genetic evaluations, CGs are conventionally treated as fixed effects to remove systematic environmental variation and to reduce potential bias arising from associations between CG and sire usage [27, 29]. Although Schaeffer [30] argued that CGs should, in principle, be treated as random effects, other studies have indicated that the advantages of random-CG formulations are most pronounced when CG levels are numerous, subclasses are small, and genetic connectedness among groups is weak [30]. In the present study, CGs were defined under a common AAA FI testing and genetic-evaluation framework, with moderate to large group sizes. Moreover, estimates of additive genetic and residual variances and EBV rankings were highly consistent across alternative CG specifications. Under these conditions, and to align with practice in beef cattle genetic evaluations, fixed CG was retained in the reference TSFH model.

#### Two-step (TS) vs. One-step (OS) approaches

The TS–OS comparison highlights that variance partitioning can differ when (i) a derived pseudo-phenotype is constructed prior to genetic analysis, versus (ii) the same energy-balance relationship is incorporated directly into the non-linear likelihood. In the TS approach, log($\:\alpha\:$) was computed from the nutritional energy-balance framework using the observed and derived input variables. Consequently, measurement noise and approximation error from upstream components can propagate into the derived trait. Depending on how this propagated uncertainty aligns with the assumed residual structure in the subsequent animal model, part of the variation may be absorbed by the residual term, but some may also be expressed as additional between-animal or between-group variation. Similar considerations have been discussed for other derived efficiency traits (e.g., ratio- or regression-based traits such as FCR and RFI), whose genetic and statistical properties depend on the (co)variance structure of their component traits and on how error is treated during trait construction [8, 31–33]. Accordingly, estimates of variance components and heritability may be sensitive to trait definition, data editing and quality control, and residual assumptions.

In contrast, the OS approach incorporates the energy-balance equation directly into the model, with EBWG treated as the observed response and *α* represented as a latent parameter. Lack of fit between observed EBWG and model-implied expectations is therefore handled through the likelihood and its residual specification, which can lead to a different partitioning of additive genetic and residual variation compared with the TS approach. The modest differences in variance components observed between TS and OS may therefore reflect differences in model parameterization and residual treatment rather than differences in the underlying genetic signal.

Despite modest differences in variance partitioning, EBV rankings were highly concordant between TS and OS in the matched **A**-matrix comparison, indicating that the main genetic signal underlying between-animal differences was largely preserved across formulations. From a breeding-evaluation perspective, the TS approach can be incorporated directly into standard linear animal models and multi-trait genetic evaluation pipelines. The OS approach instead provides an alternative statistical formulation in which the energy-balance relationship is incorporated directly without first constructing a pseudo-phenotype. Thus, the two approaches should be viewed as complementary modeling strategies rather than as providing different levels of physiological interpretation.

In this study, the OS model was implemented using the **A** matrix primarily for computational reasons. Incorporating the single-step relationship structure into the nonlinear OS model implemented in TMB would require additional matrix operations within repeated likelihood evaluations and the Laplace approximation, increasing computational and memory demands relative to the pedigree-based implementation. To provide a consistent comparison between TS and OS under the same genetic relationship structure, the **A** matrix was used for both approaches in the direct TS–OS comparison. Although single-step genomic evaluation is widely used to improve genomic prediction in routine genetic evaluations [34, 35], extension of the OS framework to genomic evaluation would require a more computationally efficient implementation of the single-step relationship structure and remains an area for future development.

### Biological interpretations and implications for breeding

The heritability of log($\:\alpha\:$) (*h*^*2*^ = 0.13 ± 0.01) under the TSFH model indicates the presence of additive genetic variation and supports the feasibility of genetic evaluation for this derived coefficient. This finding alone, however, does not establish *α* as a breeding criterion, as its practical value depends on whether it provides useful information beyond conventional growth and feed efficiency traits. As *α* was analyzed on the log scale, genetic differences correspond to proportional differences in *α* on the original scale, facilitating interpretation of relative differences among animals. The heritability estimates for ADG, DMI, FCR, RFI, and RADG from the two-trait models were within previously reported ranges [33, 36], supporting the plausibility of the estimates obtained in this study.

By construction, *α* represents estimated NE retained in growth relative to estimated NE available for growth after predicted maintenance requirements are accounted for. Its genetic relationships with conventional growth and feed efficiency traits were therefore expected to reflect some shared genetic variation. In the present population, log(*α*) was positively correlated with ADG and RADG, negatively correlated with FCR and RFI, and only weakly associated with DMI. The strong genetic correlation with FCR (− 0.80) and moderate correlation with RFI (− 0.54) indicate substantial genetic overlap with conventional feed efficiency traits. However, genetic correlations with absolute values below unity suggest that *α* is not genetically equivalent to these traits and may capture additional genetic variation. The weak genetic correlation with DMI (− 0.14) further indicates limited genetic association with FI in this population. Furthermore, the weak negative genetic correlations of *α* with subcutaneous fat thickness, together with its near-zero genetic correlation with UREA, may be consistent with differences in the energetic costs of lipid and protein deposition [12, 37], and with previous findings that more feed efficient cattle tend to have lower subcutaneous fatness [38].

Interpretation of *α* should nevertheless consider several assumptions in its derivation. The framework applies to the same maintenance-requirement equation across animals after accounting for body size and mean environmental temperature. Individual deviations from predicted maintenance requirements, which may be partly heritable [39, 40], are therefore not separately identified or explicitly accounted for in the estimation of *α*. In addition, a common MED and fixed partial efficiencies of ME utilization for maintenance and growth were assumed across animals. Although animals within a feeding test were managed on the same diet and systematic differences among tests could be partly accounted for by the CG effect, the absence of test-specific diet energy information prevented direct adjustment for variation in dietary energy density across tests. Importantly, these unmodeled sources of variation are biologically relevant and may influence *α* in interpretable directions. For example, lower maintenance costs or more efficient utilization of energy for growth would be expected to increase the amount of energy retained in growth relative to the estimated energy available for growth, thereby increasing *α*. Variation in non-growth energy demands, including activity, thermoregulation, or immune responses, may also contribute to differences in *α*. However, these processes were not directly measured in the present study, so their relative contributions to variation in *α* cannot be determined. Accordingly, *α* may provide a basis for investigating resource-allocation trade-offs, but establishing whether its additional genetic variation is associated with specific non-growth functions will require joint analysis with independently measured physiological, health, and resilience-related traits.

From a breeding perspective, *α* may provide a complementary view of efficiency by framing between-animal differences in terms of relative energy allocation toward growth rather than feed use or production efficiency alone. The substantial but incomplete genetic overlap with FCR and RFI indicates that *α* is not genetically equivalent to these traits. However, whether the additional genetic variation captured by *α* provides useful information for breeding remains to be established. Accordingly, the present results support further evaluation of *α* rather than its immediate use as an independent selection criterion. In particular, evaluating *α* jointly with health, resilience, fertility, and other economically or functionally important traits could help determine whether it provides useful information for characterizing resource-allocation trade-offs and potential correlated responses to selection. In addition, because *α* is a derived trait, its practical value will depend on the validity of the underlying assumptions and the quality of the input data, highlighting the importance of validation across populations and environments before routine implementation.

### Data constraints and future directions

A primary limitation of this study was the use of aggregated average daily DMI and limited BW measurements, which precluded the characterization of longitudinal shifts in energy allocation throughout the growth period. For future applications of this framework, longitudinal individual FI, repeated BW and body-composition measurements, and test-specific information on diet composition and energy density would provide an important foundation. These data would allow temporal changes in *α* to be characterized more accurately, improve estimation of energy retained in growth, and reduce reliance on common assumptions regarding dietary energy supply. Ideally, these records would be complemented by more direct information related to maintenance and energy expenditure, such as heat production, activity measurements, and metabolic indicators, together with independently measured health, immune, thermal-response, reproduction, and resilience-related traits. Joint genetic analyses of *α* with such traits would help determine whether the genetic variation in *α* that is not shared with conventional efficiency traits is associated with specific biological processes and whether *α* provides useful information for investigating resource-allocation trade-offs. Such information would also support evaluating the potential role of *α* in breeding objectives that balance production efficiency with other economically and functionally important traits. More broadly, provided that suitable measurements and quantitative information on energetic requirements are available, this framework could be extended beyond growth to investigate energy allocation to other biological functions.

## Conclusions

We derived and evaluated a coefficient of energy allocation to growth (*α*) based on a nutritional energy-balance framework for American Angus cattle. Although modest differences in variance partitioning were observed between the two approaches, the high concordance in EBV rankings indicates that the genetic signal was largely consistent across model formulations. The TS framework, particularly with the **H** matrix, is compatible with standard genetic evaluation procedures and provides a practical approach for genetic analysis of this derived coefficient. The *α* showed substantial but incomplete genetic overlap with conventional growth and feed efficiency traits and only a weak genetic association with DMI in the present population. These results support *α* as a derived indicator for further investigation of relative energy allocation toward growth. Future work should evaluate its relationships with health, reproduction, and resilience-related traits to determine whether *α* provides additional information for investigating resource-allocation trade-offs and, ultimately, for breeding applications.

## Supplementary Information

Below is the link to the electronic supplementary material.

Supplementary Material 1

Supplementary Material 2

## Data Availability

The data supporting the results of this article are included within the article and in its Additional files. The raw data cannot be made available, as it is property of the American Angus Association (Saint Joseph, MO, USA), and this information is commercially sensitive.
