# Supplementary material for: Genetics of energy allocation to growth in Angus cattle: trait derivation, model comparison, and relationships with feed efficiency and body composition traits
Source: Genet Sel Evol. 2026 Oct 31;58:79. doi: 10.1186/s12711-026-01102-4 (PMC13723936; doi:10.1186/s12711-026-01102-4)
Supplement: Supplementary file 1 [file 12711_2026_1102_MOESM1_ESM.docx]

**Figure S1.** Geographic distribution of phenotypic records across feed intake testing locations in American Angus cattle.

Points represent unique geographic locations based on ZIP codes, and point size and color indicate the number of phenotypic records available at each location.
