# Supplementary material for: Genetics of energy allocation to growth in Angus cattle: trait derivation, model comparison, and relationships with feed efficiency and body composition traits
Source: Genet Sel Evol. 2026 Oct 31;58:79. doi: 10.1186/s12711-026-01102-4 (PMC13723936; doi:10.1186/s12711-026-01102-4)
Supplement: Supplementary file 2 [file 12711_2026_1102_MOESM2_ESM.docx]

**Supplementary Table S1.** Descriptive statistics for phenotypic record by sex in American Angus cattle.

| Trait | Unit | Bull | | Heifer | | Steer | |
| --- | --- | --- | --- | --- | --- | --- | --- |
|  |  | **N** | **Mean ± SD** | **N** | **Mean ± SD** | **N** | **Mean ± SD** |
| Age at the start of feeding test | d | 25,987 | 283.77 ± 48.57 | 6,899 | 316.38 ± 47.12 | 2,886 | 331.52 ± 35.95 |
| Age at the end of feeding test | d | 25,987 | 354.66 ± 46.68 | 6,899 | 383.11 ± 44.12 | 2,886 | 422.67 ± 26.94 |
| Body weight at the start of feeding test | kg | 25,987 | 360.53 ± 70.45 | 6,899 | 322.13 ± 57.86 | 2,886 | 409.31 ± 61.83 |
| Body weight at the end of feeding test | kg | 25,987 | 478.39 ± 76.99 | 6,899 | 396.72 ± 63.40 | 2,886 | 580.48 ± 66.92 |
| Feeding test duration | d | 25,987 | 70.89 ± 15.40 | 6,899 | 66.72 ± 13.42 | 2,886 | 91.15 ± 20.86 |
| Dry matter intake (DMI) | kg/d | 25,987 | 10.18 ± 1.91 | 6,899 | 9.62 ± 2.46 | 2,886 | 10.72 ± 1.76 |
| Average daily gain (ADG) | kg/d | 25,987 | 1.66 ± 0.37 | 6,899 | 1.14 ± 0.35 | 2,886 | 1.88 ± 0.33 |
| Feed conversion ratio (FCR) | - | 25,860 | 6.32 ± 1.53 | 6,560 | 8.59 ± 2.39 | 2,882 | 5.85 ± 1.24 |
| Residual feed intake (RFI) | kg/d | 25,987 | -0.01 ± 1.46 | 6,899 | 0.42 ± 1.94 | 2,886 | -0.87 ± 1.53 |
| Residual average daily gain (RADG) | kg/d | 25,987 | 0.04 ± 0.35 | 6,899 | -0.24 ± 0.34 | 2,866 | 0.19 ± 0.31 |
| Ultrasound ribeye area (UREA) | cm² | 21,245 | 80.37 ± 10.56 | 4,438 | 61.40 ± 9.94 | - | - |
| Ultrasound rib fat depth (UFATrib) | cm | 21,264 | 0.65 ± 0.24 | 4,477 | 0.63 ± 0.29 | - | - |
| Ultrasound rump fat depth (UFATrump) | cm | 21,247 | 0.73 ± 0.25 | 4,472 | 0.75 ± 0.30 | - | - |
| Ultrasound intramuscular fat (UIMF) | % | 21,263 | 3.99 ± 1.03 | 4,471 | 5.47 ± 1.28 | - | - |
| Yearling hip height (YHP) | cm | 10,909 | 126.98 ± 4.55 | 3,503 | 122.89 ± 4.73 | 211 | 127.00 ± 3.85 |
| Energy allocation coefficient ($\alpha$) | - | 25,650 | 0.81 ± 0.25 | 6,747 | 0.50 ± 0.20 | 2,879 | 1.02 ± 0.32 |
| Log transformed energy allocation coefficient(log$(\alpha)$) | - | 25,650 | -0.26 ± 0.31 | 6,747 | -0.76 ± 0.38 | 2,879 | -0.02 ± 0.29 |
